# Violence against healthcare workers in Nepal: a system dynamics perspective on a growing crisis

**DOI:** 10.3389/fpubh.2025.1615231

**Published:** 2025-07-04

**Authors:** Animesh Ghimire

**Affiliations:** ^1^Faculty of Medicine, Nursing and Health Science, Monash University, Melbourne, VIC, Australia; ^2^Sustainable Prosperity Initiative Nepal, Kathmandu, Nepal

**Keywords:** system dynamics, workplace violence, healthcare workers, resource scarcity, impunity, physical abuse, mobile applications, waiting lists

## 1 Introduction

Violence against healthcare workers (HCWs) is increasingly recognized as a global public health emergency, eroding not only the safety of frontline providers but also the integrity of healthcare systems themselves (1). The pervasiveness of this issue is underscored by extensive international research. A 2024 narrative review by O'Brien et al. (2) highlights that despite decades of interventions, workplace violence (WPV) is worsening globally in both severity and frequency, driven by factors like long wait times, poor communication, and organizational failures. This is corroborated by the large-scale ViSHWaS global survey, which found that over half (55%) of HCWs had experienced firsthand violence, with patients or their families being the perpetrators in the majority of cases (3). The consequences are severe, contributing to burnout and a diminished workforce, a dynamic influenced by factors ranging from systemic pressures to individual characteristics like emotional resilience (4). In Nepal, the situation has reached a critical juncture, as escalating physical assaults and incidents of hospital property destruction have exposed deep-seated systemic pressures, ranging from chronic resource constraints to inadequate communication channels (5–7). While national data is limited, localized studies from urban teaching hospitals indicate that nurses often bear a disproportionate burden of this violence compared to doctors, particularly verbal abuse (8, 9). Although stricter legal penalties have been introduced in an attempt to deter attacks, they alone have not curbed the violence, signaling that punitive measures do little to address the root causes (7).

Traditional approaches to HCW violence often emphasize punitive or incident-based responses, yet frequently fail to capture the complex, feedback-driven dynamics underlying such events. To address this gap, this opinion piece employs system dynamics modeling to dissect the interconnected factors fueling HCW violence in Nepal (10). Beyond categorizing individual incidents, this approach illuminates how resource scarcity and prolonged waiting times—often perceived by patients as unjust—reinforce frustration and aggression. By mapping these reinforcing loops, I move past a purely descriptive narrative to propose a targeted intervention: a novel mobile application designed to enhance transparency around wait times, manage patient expectations, and improve communication. This digital tool is conceptualized not as a panacea, but as a strategic instrument to disrupt key feedback loops, thereby mitigating a primary trigger of aggression. Through this systemic lens, I argue that sustainable change in Nepal's healthcare environment demands multifaceted solutions capable of transforming an under-resourced and tension-laden system into one that is safer and more equitable for providers and patients.

## 2 The vicious cycle: a system dynamics model of violence

**Figure 1:**
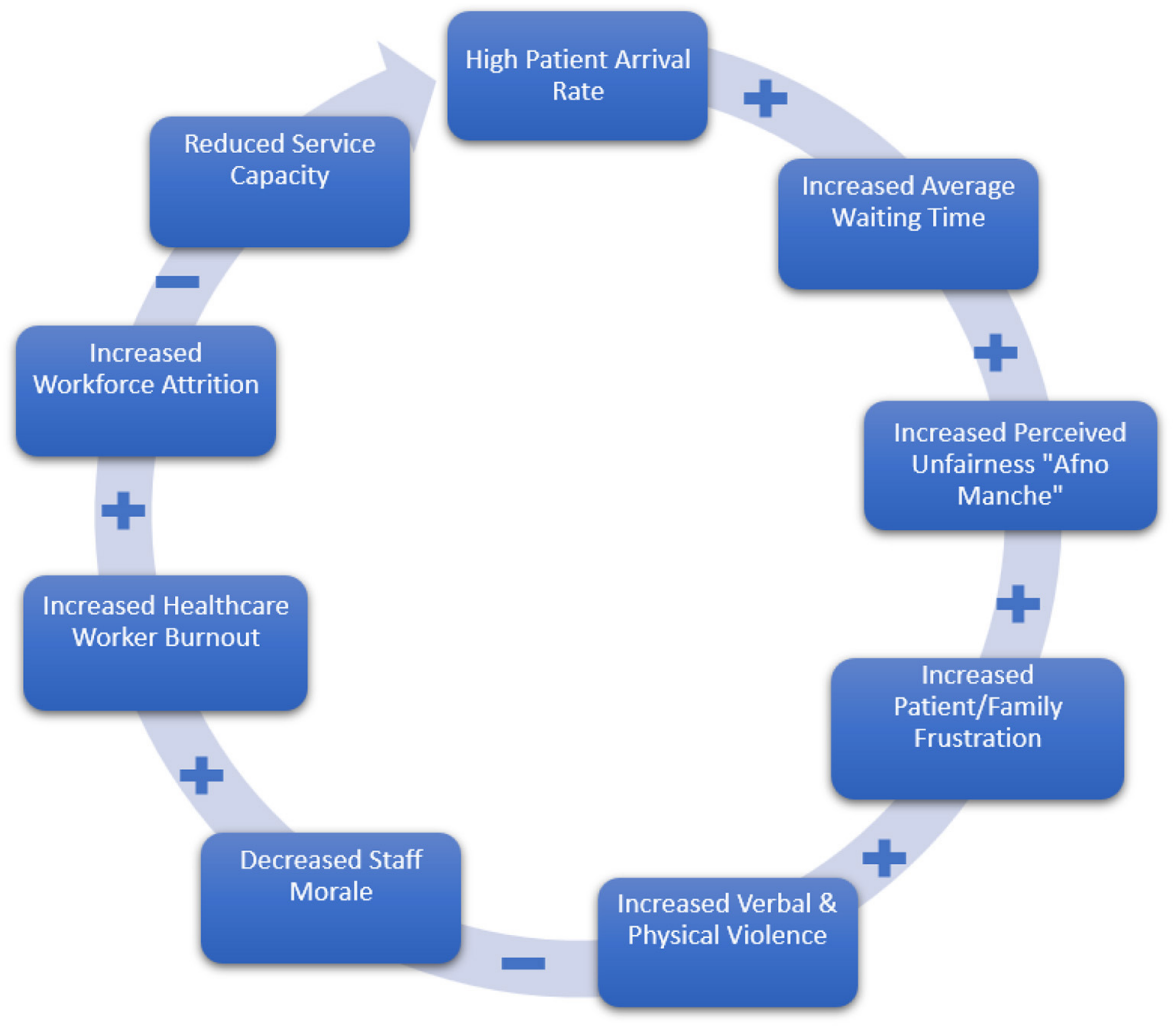
The core vicious cycle driving violence against healthcare workers in Nepal. This diagram illustrates the primary feedback loop where high patient arrival rates lead to increased average waiting times, fueling patient/family frustration. This frustration can escalate to verbal and physical violence, which degrades staff morale, increases HCW burnout and workforce attrition, and ultimately reduces service capacity, thereby further prolonging waiting times. While this diagram highlights the core operational and workforce cycle, it is amplified by additional reinforcing loops involving personal connections (“Afno Manche”), political impunity, and defensive medicine practices, as detailed in the subsequent discussion. Positive (+) and negative (–) signs indicate the direction of causal relationships.

Violence against HCWs in Nepal is not an isolated phenomenon; rather, it emerges predictably from the pressures and constraints of an overburdened healthcare system. Figure 1 presents a simplified, conceptual system dynamics model focusing on the core vicious cycle that perpetuates this crisis. This primary loop illustrates how high patient volumes and resource scarcities escalate the risk of violence, ultimately harming both HCWs and patient outcomes. This core mechanism is then amplified by several interconnected reinforcing feedback loops involving cultural practices, political dynamics, and the direct impact of violence on healthcare professionals.

### 2.1 Core loop (the engine of violence)

At the heart of this model lies the interplay between **Patient Arrival Rate**, **Service Capacity**, and **Average Waiting Time**. Chronic resource shortages—encompassing limited staffing, bed availability, and equipment—mean that high patient arrivals routinely overwhelm the system (11). This imbalance directly prolongs waiting times (+), which significantly heightens patient and family frustration (1, 11, 12). Escalated frustration translates into a greater likelihood of verbal and physical violence (+) against HCWs (13), thereby reducing service capacity (–) as morale and productivity decline. This loop functions as a classic reinforcing feedback structure, where systemic strain leads to patient frustration, which in turn increases violence, further reducing staff capacity and prolonging wait times.

### 2.2 Personal connection (“Afno Manche”) and the erosion of fairness

The deeply entrenched “Afno Manche” system—which translates to “one's own people” and signifies a culture of favoritism based on personal or social networks—constitutes another powerful feedback loop (1). Individuals with connections experience shorter waiting times (–), while those lacking such connections perceive heightened inequity, exacerbating their frustration (+). This sense of unfairness further reinforces the conditions conducive to violence against HCWs. Beyond fostering resentment toward providers, this dynamic also fuel inter-patient tensions, creating a more broadly contentious and stressful environment within the healthcare facility.

### 2.3 Political patronage and impunity

Political influence compounds the problem through a *Climate of Impunity* (1, 5). Perpetrators connected to political networks often evade legal repercussions, which emboldens further aggression (+). The resulting spike in violence not only jeopardizes staff safety but also validates the precedent of unchecked hostility.

### 2.4 The toll on healthcare providers

Acts of verbal and physical violence exact a significant toll on HCWs themselves, forming a loop that diminishes overall service capacity (1, 5, 12). Violence erodes **Staff Morale** (–) and drives up **Burnout** (+), which, in turn, accelerates **Workforce Attrition** (+). This aligns with alarming trends of health workforce migration from Nepal, where poor working conditions, including the threat of violence, contribute to a significant “brain drain” of skilled professionals (14, 15). The loss of skilled personnel not only places additional strain on the remaining staff, perpetuating system-wide fragility, but also severely disrupts the continuity of patient care, leading to poorer health outcomes.

### 2.5 Defensive medicine

Finally, mounting violence propels **Defensive Medicine** (+). As clinicians seek to safeguard themselves from accusations or attacks, they order additional tests and referrals, consuming more time and resources (6, 11, 12). This behavior further diminishes effective service capacity (–) and intensifies overcrowding, thus feeding back into the core loop of waiting time and frustration.

### 2.6 Implications

By mapping these interconnected feedback loops, the system dynamics model sheds light on how disparate factors—resource deficiencies, cultural norms, political patronage, and HCW burnout—reinforce one another. Effective interventions, therefore, require addressing systemic drivers rather than mere symptoms. Understanding these complexities is essential for designing targeted strategies that reduce violence and protect the workforce, which is essential for delivering quality care.

## 3 A mobile intervention: targeting systemic leverage points

The primary objective of the proposed intervention is to disrupt the core feedback loops driving violence by enhancing systemic transparency and managing patient expectations. To achieve this, a novel mobile application, “Sustha Samaya” (which translates to “Healthy Time”), is proposed. This intervention is designed to target the critical leverage points of excessive waiting times and communication breakdowns, thereby fostering a more predictable and less frustrating healthcare experience.

### 3.1 Aligning “Sustha Samaya” features with system dynamics

The core features of “Sustha Samaya” are strategically designed to intervene in the identified feedback loops:

- Mitigating waiting-time frustration:
  ○ The application would display “*real-time”* waiting time estimates across various departments (e.g., emergency, outpatient, specialty clinics). This directly addresses the positive feedback loop where prolonged “*Average Waiting Time”* fuels “*Patient/Family Frustration”* by reducing uncertainty and managing expectations.
  ○ Additionally, by showing facility capacity information—such as the number of on-duty doctors or open beds—patients can make informed decisions, potentially diverting patient flow from already overstretched facilities and thereby indirectly acting on the “*Patient Arrival Rate”* variable at specific locations and easing the “*Service Capacity”* strain.
- Countering perceived unfairness and enhancing communication:
  ○ Recognizing how the “*Afno Manche”* system contributes to “*Perceived Unfairness”* and thus “*Patient/Family Frustration*,” the app would outline transparent triage processes. This would be operationalized, for instance, by displaying a simplified, color-coded urgency scale (e.g., Red-Immediate, Yellow-Urgent, Green-Stable) alongside brief, text-based explanations of the criteria used for prioritization. This aims to disrupt the loop where lack of clarity amplifies frustration by providing open access to prioritization guidelines.
  ○ An anonymous feedback mechanism—whereby user-identifying data is stripped before feedback is aggregated into thematic reports for hospital administration—allows patients and families to voice their concerns. This provides a constructive outlet that can reduce the build-up of unresolved “*Patient/Family Frustration”* before it escalates to “*Verbal and Physical Violence*.”
  ○ By providing easily accessible, context-relevant medical information (Health Literacy Support)—delivered through simple Nepali text, infographics, and potentially short video clips—the app aims to improve understanding of healthcare procedures, thereby reducing anxiety and mistrust that can fuel the “*Patient/Family Frustration”* to “*Verbal and Physical Violence”* pathway.

### 3.2 Feasibility, precedents, and next steps

Nepal's steadily rising mobile phone penetration rate underpins the viability of “Sustha Samaya” (16). While this specific application is novel in its direct targeting of violence-related system dynamics in Nepal, the use of mobile health (mHealth) interventions to improve patient experience, manage hospital workflows, and enhance communication is well-documented globally. For instance, mHealth applications in India have been deployed to streamline appointment scheduling and reduce outpatient waiting times, demonstrating the potential to alleviate key stressors in high-volume settings (17, 18). Similarly, initiatives in several sub-Saharan African countries have leveraged mobile technology to bridge communication gaps between patients and healthcare providers, leading to improved patient satisfaction and more efficient care navigation (19, 20). These precedents suggest that a thoughtfully designed mobile intervention, sensitive to local context, can be a feasible component of broader strategies to mitigate workplace stress and potential conflict in healthcare.

Although challenges—such as ensuring data accuracy, reliable internet connectivity, and varying levels of digital literacy—persist, these obstacles can be addressed through a carefully designed implementation strategy. This would involve phased pilot testing in both urban and rural settings to assess contextual differences, incorporating multilingual support where necessary, and utilizing simple iconography and visual aids to overcome potential digital literacy barriers. Crucially, this intervention is not intended as a standalone solution; instead, it aims to disrupt the core loops that contribute to violence by enhancing communication and transparency. When paired with broader systemic reforms, such as increased resource allocation and consistent legal enforcement, “Sustha Samaya” may offer a context-sensitive, scalable strategy to curb HCW violence and foster a more equitable healthcare experience.

## 4 Discussion

Punitive measures are insufficient to curb violence against HCWs because, as this analysis demonstrates, the phenomenon is rooted in systemic failure. The system dynamics model in Figure 1 offers critical insights into why purely punitive measures—such as harsher legal penalties—have had limited success (7). These measures address only a single node in a complex, interlocking network of factors. Without tackling underlying drivers—resource scarcity, breakdowns in communication, entrenched cultural norms such as “Afno Manche,” and political interference—violence is likely to persist and potentially worsen (21). This echoes broader public health research that has repeatedly shown violence to be a symptom of deeper systemic weaknesses in healthcare (22). Indeed, an umbrella review by Rossi et al. (23) substantiates this systemic framing on a global scale, summarizing that common factors contributing to workplace violence in healthcare settings include high stress levels, prolonged work hours, dissatisfaction with treatment, excessive waiting times, and high medical costs, often positioning patients and their relatives as the primary offenders. The review further notes the pervasive issue of underreporting and the severe consequences of violence—such as diminished job satisfaction, burnout, and detrimental effects on patient care—all of which align with the structural issues identified within the Nepali context. For instance, studies in India highlight overcrowding, long wait times, and insufficient communication as triggers for aggression toward providers (24), while work in sub-Saharan Africa underscores how limited resources and weak governance directly threaten HCW safety (25). Thus, the Nepali context exemplifies a larger, global pattern of healthcare violence fueled by multifaceted systemic deficiencies.

## 5 Recommendations

Based on this systemic analysis, achieving sustainable change necessitates a comprehensive strategy that extends beyond any single intervention. Key recommendations include:

- Addressing resource scarcity: significant and sustained investment in healthcare infrastructure and human resources is essential, especially in underserved rural areas. This includes expanding the workforce of doctors, nurses, and support staff, along with ensuring adequate equipment and supplies.
- Strengthening security and accountability: stronger security measures and stricter accountability frameworks are crucial. Healthcare facilities must be made safer, while legal mechanisms ensure that perpetrators face consequences irrespective of political connections (1, 5–7).
- Fostering a culture of respect: a cultural shift toward respect and realistic expectations is vital. Public awareness campaigns should clarify the realities and limitations of the healthcare system while underscoring respectful communication. In parallel, HCWs need training in communication and de-escalation skills to manage tense situations more effectively.
- Countering “Afno Manche” favoritism: this demands a long-term commitment to transparent triage protocols, clear patient-prioritization criteria, and the promotion of ethical conduct among healthcare professionals to build trust, equity, and consistency in care delivery.

## 6 Limitations

This opinion piece utilizes a conceptual system dynamics model, which, while effective for illustrating complex feedback structures, has inherent limitations. The model is a qualitative abstraction intended to map relationships rather than a quantitative simulation to predict outcomes. The connections and polarities are based on a synthesis of existing literature and may not capture the full heterogeneity of experiences across all healthcare settings in Nepal.

Furthermore, the proposed “Sustha Samaya” mobile application, while grounded in the model's logic, faces practical hurdles. Its feasibility is contingent upon overcoming challenges related to data integration from disparate hospital systems, ensuring reliable internet connectivity, and addressing varying levels of digital literacy among the patient population. The behavioral impact also warrants consideration; while the app is designed to reduce frustration, it cannot be assumed that access to information will uniformly alter entrenched behaviors without complementary social and educational interventions.

## 7 Conclusion

The system dynamics driving violence against Nepal's healthcare workers are clearly demonstrated by this analysis: a predictable confluence of resource scarcity, prolonged waiting times, entrenched systems of favoritism like “Afno Manche,” and pervasive impunity. Technological interventions, particularly mHealth applications, may offer temporary relief by addressing friction points; the proposed “Sustha Samaya” app is conceptualized as one such tool focused on waiting and communication, yet it cannot fundamentally alter the trajectory of a system under such strain. True transformation requires grappling with the uncomfortable reality that this violence is not an aberration, but an expected output of the current systemic configuration. This underscores the need to critically examine the locus of responsibility for systemic reform and to consider the long-term viability of a healthcare system where providers consistently operate under the threat of violence.
